# Challenges of implementing the accreditation model in military and university hospitals in Iran: a qualitative study

**DOI:** 10.1186/s12913-020-05536-4

**Published:** 2020-07-29

**Authors:** Leila Vali, Mohammad Hossein Mehrolhasani, Saeid Mirzaei, Nadia Oroomiei

**Affiliations:** 1 Environmental Health Engineering Research Center, Kerman University of Medical Sciences, Kerman, Iran; 2 Research Center for Health Services Management, Institute for Futures Studies in Health, Kerman University of Medical Sciences, Kerman, Iran; 3 Department of Health Management, Policy and Economics, School of Public Health, Bam University of Medical Sciences, Bam, Iran

**Keywords:** Accreditation model, Military hospitals, University hospitals, Challenge, Iran

## Abstract

**Background:**

The aim of this study was to present challenges of implementing the accreditation model in university and military hospitals in Iran.

**Methods:**

In this qualitative study, purposive sampling was used to select hospital managers and implementers of the model working in 3 hospitals affiliated to Kerman University of Medical Sciences and in 3 military hospitals in Kerman, Iran. A total of 39 participants were interviewed, and semi-structured questionnaires and thematic analysis were used for data collection and analysis, respectively.

**Results:**

In this study, 5 major codes and 17 subcodes were identified: (1) perspectives on accreditation model with 5 subcodes: a difficult and time-consuming model, less attention to the patient, accreditation as a way of money acquisition, not being cost-effective, and accreditation means incorrect documentation; (2) absence of appropriate executive policy, with 3 subcodes: lack of financial funds and personnel, disregarding local conditions in implementation and evaluation, and absence of the principle of unity of command; (3) training problems of the accreditation model, with 2 subcodes: absence of proper training and incoordination of training and evaluation; (4) human resources problems, with 3 subcodes: no profit for nonphysician personnel, heavy workload of the personnel, and physicians’ nonparticipation; (5) evaluation problems, with 4 subcodes: no precise and comprehensive evaluation, inconformity of authorities’ perspectives on evaluation, considerable change in evaluation criteria, and excessive reliance on certificates.

**Conclusions:**

This study provided useful data on the challenges of implementing hospitals’ accreditation, which can be used by health policymakers to revise and modify accreditation procedures in Iran and other countries with similar conditions. The accreditation model is comprehensive and has been implemented to improve the quality of services and patients’ safety. The basic philosophy of hospital accreditation did not fully comply with the underlying conditions of the hospitals. The hospital staff considered accreditation as the ultimate goal rather than a means for achieving quality of service. The Ministry of Health and Medical Education performed accreditation hastily for all Iranian hospitals, while the hospitals were not prepared and equipped to implement the accreditation model.

## Background

Accreditation and continuous quality improvement is an inseparable part of health service activities. Health care accreditation programs started in the 1970s and accreditation organizations have been established and developed since then [1]. The Joint Commission on Accreditation of Healthcare Organizations (JACHO) and the Australian Council of Healthcare Standards (ACHS) are the 2 most famous accreditation systems in the world. JACHO emphasizes on providing the best practice by using appropriate quality indicators and identifying and preventing health care injuries by designing standards. The ACHS program emphasizes on clinical care improvement, self-improvement, and specialists involvement in quality activities [2]. Many countries have implemented health care accreditation and have achieved different results, and many others have implemented accreditation without strong evidence to prove that accreditation is the most appropriate source for improving the quality of health services [3]. Review of the literature on the impact of accreditation on quality of care does not provide strong evidence [4].

Devkaran et al. argued that there is little empirical evidence on the benefits of accreditation [5], while a large number of countries, including the United Arab Emirates, use accreditation as a means of ensuring the quality of health services [6]. The findings of the systematic review by Nicklin et al. revealed several positive benefits to accreditation; however, the study was not accurate enough [7]. Overall, the evidence for the credibility of the accreditation model is scattered, especially in the Middle East [8].

In Iran, the government is responsible for providing health care services to the public [9], which is done by the Ministry of Health and Medical Education (MOHME). The MOHME, as the public health custodian, has always attempted to apply the most efficient strategies to achieve health objectives. This is achieved by identifying the individuals and society’s health needs and risk factors and benefiting from new scientific methods of policy planning and decision-making [10]. To provide safe and efficient services, during the last 2 decades, Iran’s Deputy of Treatment of the Health Ministry has taken effective steps to organize this scope by benefiting from hospitals’ evaluation guide [11]. One of these steps is implementation of the accreditation model in hospitals.

There has been a need to improve the quality of health services in Iranian hospitals. Some hospitals voluntarily used ISO and EFQM industrial models for evaluation. The growing recognition of credible hospital accreditation around the world, especially in EMRO countries such as Lebanon, has encouraged some Iranian hospitals to seek international accreditation programs. Given that the accreditation model has been developed specifically for the health sector, the MOHME decided to develop a national accreditation model for Iranian hospitals [12]. Few countries have conducted studies on accreditation models, including France, Egypt, the United States, Canada, Australia, and Lebanon. A literature review was conducted and hospital standards of these countries were reviewed. Then, expert focus groups were held with regards to quality promotion and accreditation [13]. Finally, accreditation standards of Iranian hospitals were developed. The accreditation model in Iran is more similar to that in the United States. The 3 components of structure, process, and output/outcome were considered in hospital standards. However, lower weight was given to the output/outcome standards [2]. The initial goals of the accredited program were as follow: use more comprehensive structural, procedural, and outcome standards to improve the quality and safety of hospital services; better respond to patient’s needs; improve hospital status and key performance indicators; reduce costs; and enhance patient satisfaction [11].

The Control and Accreditation Office of the MOHME, in line with its main responsibility, which is to control health care services to ensure their quality and safety and to update scientific evidence-based feature, has substituted the evaluation of health care centers with accreditation model since 2007 [11]. For hospital accreditation, the MOHME announces the standards and the evaluation time to the hospitals, obliging them to take measures and upload documentation of their operations in the National Hospital Information Accreditation System. Then, the evaluation time is announced to hospitals after documents have been reviewed by universities of medical sciences. At the time of the evaluation, the evaluators at universities of medical sciences, most of whom have clinical experience, visit the hospitals and evaluate their performance according to the checklist. Hospitals’ tariffs depend on the rating status after evaluation [11].

The first edition of the Iranian hospital accreditation standards was developed in 2010 in the form of 37 hospital wards and 8104 metrics, which was announced to hospitals across the country. Standards were more of structural and process type, and there were a limited number of output/outcome standards. Also, the evaluation team comprised 20 to 25 surveyors [14]. In 2014, hospital accreditation standards were revised and the second edition of the hospital accreditation standards was compiled in 36 hospital wards and 2157 metrics [11].

Hospital Joint Measures in the form of enterprise-centric integrated guidance were expressed in the management and leadership unit. A number of similar standards were separated from the various sections and cited as common clinical and nonclinical standards [13]. Then, the Iranian hospital accreditation system was revised in 2017, and accreditation standards were developed in the form of 8 scoops, 248 standards, and 903 metrics [11].

Implementation of the accreditation model, like any other model, has some challenges whose identification can help better implement the model, improve policymaking, and execute similar models in Iran as a developing country. Moreover, Iran’s experience in implementing the accreditation model can also be useful to other developing countries.

Since only few studies have been conducted on accreditation implementation in university and military hospitals in Iran, this study aimed to present challenges of implementation of the accreditation model in these hospitals to the health system authorities and policymakers. In addition, this study was conducted in Kerman, which is the largest city of the largest province of Iran. In Kerman, hospitals are supervised by Kerman University of Medical Sciences, and there are 2 types of hospitals: academic and military. Academic hospitals are managed by medical sciences universities, and military hospitals provide health care services to military forces. Providing high quality services to military personnel in military hospitals is highly important [15, 16].

## Methods

In this qualitative case study, which was conducted in 2019, purposive sampling was used to select the participants. Those who had knowledge about hospital accreditation and those responsible for performing accreditation in hospitals were included in the study. There are 4 academic hospitals and 3 military hospitals in Kerman, which were included in this study, and those staff who met the inclusion criteria entered the study.

Overall, 23 staff of military hospitals and 16 of academic hospitals, including hospitals’ heads, matrons, accreditation supervisors, all supervisors of clinical wards, and accreditation executive experts, were interviewed. Sampling was continued until data saturation, with 39 interviews. Of the interviewees, 20 were women and the rest were men. Also, 12, 10, and 17 interviewees held PhD/ MD, MSc, and BSc degrees, respectively. Moreover, the researcher did not have any relationship with the participants prior to study commencement.

A meeting was held with members of the project team to formulate the research questions. A semi-structured questionnaire with open questions was used for data collection. The interview guide developed for this study is provided as Additional file 1.

One member of the research team conducted the interviews (N.O). Also, data were collected through 15–40 min face-to-face interviews, recorded by a tape recorder in hospitals. Only participants and the researcher were present at the time of the interview. During the interview memos were written.

Data were analyzed using thematic analysis. First, each interview was typed at the earliest time possible after the interview by one member of the research team (N.O). Next, the typed text was repeatedly studied by 2 members of the research team (N.O, L.V), and subcodes were identified. Then, the subcodes were studied several times and the main codes were extracted and sorted. MAXQDA software version 10 was used for data analysis and coding. Then, the codes and subcodes were compared by 2 members of the research team (MH.M, S.M) and differences were discussed in a meeting attended by all members of the research team and finalized.

The validity and reliability of the data were determined using Lincoln and Guba’s criteria [17]. The credibility of the results was guaranteed by allocating sufficient time to data collection and interpretation to understand the participants’ perspectives. A diverse and heterogeneous sample of interviewees was used for transferability, which is equivalent to generalization in quantitative researchers. To ensure credibility and conformability, the implemented transcripts of the interviews and extracted themes were provided to the research participants and their comments were used.

### Ethics, consent, and permissions

This study was approved by the Ethics Committee of Kerman University of Medical Sciences. Prior to conducting the interviews, the research team explained the aim and process of the study to the participants and obtained written informed consent from all of them.

## Results

The results of this study were categorized into 5 major codes and 17 subcodes.

### Perspectives on the accreditation model

This major code showed participants’ perspectives on the accreditation model. Overall, participants had a negative viewpoint about the hospital accreditation model and believed that this model added to workloads of hospital staff. The participants stated that the purpose of the accreditation model was to improve working conditions through process modifications, so that performing tasks according to defined standards would be the easiest way to perform their duties in the hospital; however, this aim was not achieved.

#### A difficult and time-consuming model

The participants believed that the accreditation model was idealistic and time-consuming and did not fit the conditions of the hospitals. In fact, without essential foundations, continuous implementation of this model did not lead to achievement of its goals.

*"Considering the heavy workload and lack of training, this model is unrealistic for our hospitals. We imported this model without receiving sufficient training and consideration, just like other technologies that had been imported."* [3]

#### Less attention to the patient

Participants demonstrated that paying great deal of attention to other parts of this model removes the patient from the center of attention, as this model involves the staff in documentation rather than patient care.

*"This model does everything except improving patients’ conditions. According to this model, this place is a school and we are students who need to do our homework. Also, the post model conditions not only did not improve patients’ conditions but also worsened them.”* [14]

#### Accreditation as a way of money acquisition

According to one participant, the accreditation model has become a way for money-making instead of a model for quality improvement. Hospitals do not provide training prior to the accreditation process, and this is while hospitals should invite educators and spend money on training. The hospitals invite educators from university faculty members, the evaluation team members, or MOHME’s medical deputy staff who have information about accreditation, but they ask for money to hold training classes for hospitals.

*"Accreditation was a way of money acquisition for some people involved. It was implemented just for the benefit of several people. We need training, but we are told we should pay first. It is especially worse for military hospitals, which are victims to negligence of the Ministry of Health."* [21]

#### Not being cost-effective

A significant number of interviewees believed that the accreditation model is not cost-effective and it is only a waste of resources and does not result in any changes in hospital performance.

*“These programs waste the personnel’s’ time, money, and energy. Actually, after using the model, no change has occurred in patients' satisfaction rate."* [2]

#### Accreditation means incorrect documentation

According to one respondent, a quality improvement project with the help of the accreditation model is a bureaucratic process which can lead to incorrect documentation.

*“We (the personnel) were suddenly involved without any prior preparation. We made documentation without prior training, which led to preparing an operational plan without really knowing what it was. So, I made documentations incorrectly."* [39]

*"For example, one of the documents we need to provide in the accreditation model is that doctors must provide full explanation to patients, write down the descriptions they have given to patients in a form, and stamp and sign it. However, in practice, doctors do not give patients complete and accurate explanations, and the forms are completed by the nurses, and the doctors only stamp and sign the form. Did the doctor provide a full explain to the patient? The answer is no. However, the document indicate the doctor provided a complete explanation to the patient."* [8]

### Absence of appropriate executive policy

According to participants, one problem with the accreditation model is lack of proper policymaking in implementing this model. To implement projects successfully, adequate foundations, including sufficient resources and attention to the conditions of the place of implementation, are required.

#### Lack of financial funds and personnel

This subcode indicated that one challenge of accreditation model was lack of sufficient financial funds for implementation. The hospitals faced budget shortages and were unable to cover the costs of implementing the plan, including the cost of printing paper forms to be completed and provided as hospital documentation.

*"We even have a problem for paying for the printing and copying costs of the accreditation model forms. How can we pay for better training and evaluation?"* [8]

#### Disregarding local conditions in implementation and evaluation

Insufficient infrastructure has been mentioned as a challenge. Since Iran is a vast country with various climatic conditions and facilities, one challenge of nationwide implementation of the accreditation model is disregarding local conditions of each region in the model, especially in deprived areas where hospitals do not even have enough staff. In fact, many interviewees believed that the MOHME has only considered Tehran’s hospitals and not the conditions of hospitals in other provinces of Iran.

*"This project is raw; it was run for the whole country while not adjusted to different conditions in various regions of the country. While the number of my employees is limited and each nurse does the job of 4, how can I impose this project on them and ask them to write an operational plan?"* [21]

#### Absence of the principle of Unity of command

Participants stated that one challenge of implementing the accreditation model in military hospitals is the absence of the principle of unity of command. These hospitals are affiliated to both the MOHME and the related military organization (eg, the Islamic Republic Revolutionary Guard, Army, and Police), which has put more pressure on the staff of these hospitals.

*“We have to deal with military laws and the Ministry of Health orders and models worsen the situation. Sometimes, we are really confused about which part we should consider? The military unit or university?”* [18]

### Training problems of the accreditation model

Training problems refer to all obstacles of teaching this model to hospitals’ staff. According to participants, these problems have prevented a proper understanding of the model’s philosophy and have led to its inappropriate implementation.

#### Absence of proper training

This subcode refers to insufficient and incorrect training as a background in the past and during university education and implementation. According to participants, cascading trainings were organized to be transmitted from the Control and Accreditation Office of the MOHME to universities of medical sciences (UMS) and then to hospitals. However, these trainings were provided in a short time and had a low quality.

#### Incoordination of training and evaluation

It refers to the difference between accreditation training courses and evaluation criteria and indices. Interviewees noted differences between what was trained by the UMS and what was evaluated. In fact, the training did not conform to the measures requested in the checklists.

*"Whatever they taught us completely differed from what they wanted from us and what was evaluated.”* [28]

*"They gave us a book and told us to pay for the exam and get the certificate. All those who paid the money, even those who wrote nothing on their paper, got the certificate."* [35]

### Human resource problems

Interviewees noted that staffing problems were the biggest obstacle in implementing the accreditation model. In this model, nurses are responsible for everything, while all rewards go to physicians.

#### No profit for nonphysician personnel

Interviewees believed that the model did not include any remuneration for nonmedical personnel and that all responsibility for the implementation was on the shoulders of nonmedical personnel, which reduced the motivation of the staff and led to their negative viewpoint towards the model.

"*The money goes to the physicians; what benefit does this extra responsibility bring me? All troubles are for me. If the hospital score is low, they punish me, but if it goes up, money and rewards go to others."* [33]

### Heavy workload of the personnel

Interviewees noted that the accreditation model increased personnel’s workload and decreased the quality of their performance. This situation is even worse for hospitals with insufficient staff.

*"It is the worst plan ever which imposes great psychological pressure on personnel. I should always keep the patient waiting because I am busy filling out forms."* [29]

#### Physicians’ nonparticipation

Although the model specifies the actions to be taken by physicians and they should fill out forms, interviewees noted that these tasks were done by other personnel, especially nurses; the forms were filled out by the nurses and eventually signed by physicians.

*"This project is for the nurses not the physicians. Nurses write it and physicians sign it. Accreditation should start from physicians."* [38]

### Evaluation problems

This code addresses issues related to the evaluation process performed by the accreditation model evaluators.

#### No precise and comprehensive evaluation

According to this subcode, one of the evaluation problems of this model is inattention to all aspects of performance, and this is another cause of incorrect documentation. Interviewees stated that evaluators only reviewed the documentation and did not evaluate the patients’ condition. Processes and actions in hospital accreditation forms that have been documented were not double-checked with patients to ensure their correctness and lack of fabrication. Therefore, hospital staff do not have the incentive to implement the model properly, and given the difficulties involved in implementing the model, they prefer to engage in incorrect documentation. In fact, the tracer methodology which traces the real services that patients receive, such as patient journey surveys, can be helpful in monitoring the effectiveness and efficiency of the model. However, this part has been neglected.

*"At evaluation time, the assessors just see the papers not the patient, so I spend a lot of time to complete these forms. The evaluator just sees documents and does not realize the amount of time and effort we put into all this. Why should I work so hard and honestly? I can just present a document."* [21]

#### Inconformity of authorities’ perspectives on evaluation

This subcode refers to the difference of perspectives on evaluation and absence of a clear source for solving ambiguities concerning evaluation. Interviewees indicated that evaluation checklists were not transparent. The lack of transparency in the checklists led to personal preferences and disagreements among the evaluators.

*“The most important problem related to authorities is evaluators’ disagreement. The members of the evaluation team did not have sufficient and similar information. They did not agree with each other, and there was a great difference in their perspectives.”* [26]

#### Considerable change in evaluation criteria

This subcode refers to changes of evaluation criteria during one period and causes a change in the whole process of accreditation implementation in hospitals. Interviewees mentioned that in the old version, standards were announced by the MOHME for 36 hospital wards and 2157 measures. Standards were more of a structural and process type. Then, 1 month before the evaluation, the new version was announced by the MOHME. Standards were announced in 8 scopes, 248 standards, and 903 measures. Common measures of hospital wards were integrated in the format of management and leadership unit. The combination of the measures also had an impact on the evaluation process. Related metrics were reviewed in all previous sections of the accreditation in the previous version and reviewed in the new version in limited wards of the hospital at the discretion of the evaluator. In addition to structural and process standards, a small number of outcome standards were added. Also, making changes in evaluation indices and measures in a short time before the evaluation was unpleasant for interviewees. Changing the process so close to evaluation time, although resulted in fewer standards, discouraged the staff.

*“Sudden changes of criteria 2 months before the evaluation was like a nightmare for us. There is no standard form. We should adopt ourselves to interpretations of our managers.”* [20]

#### Excessive reliance on certificates

This subcode refers to the training courses that the staff must pass before starting the accreditation assessment and present the certificate at the time of the assessment. In fact, excessive emphasis of checklists on considering certificates with no attention to learning or actions taken in the field is a challenge in this model. Excessive reliance on certificates means to present certificates only to obtain a score with no change of behavior. Poor learning causes different problems, such as incorrect documentation in the implementation stage, as some of the evaluation checklist indicators include completing training courses by hospital staff.

Evaluators only check the course certificate, and in reality no behavioral change occurs in the personnel as the result of the implementation of the model. However, the tracer methodology can be helpful in clarifying what happens in practice, not in theory, in hospitals.

## Discussion

The first main identified code was the perspectives of interviewees about accreditation. This major code included the following 5 subcode: (1) a difficult and time-consuming model; (2) less attention to the patient; (3) accreditation as a way for money acquisition; (4) not being a cost-effective accreditation model; and (5) accreditation means incorrect documentation. Thus, it should be noted that the subcodes are based on the opinions of the interviewees, which can be due to the implementation problems of this model in Iran.

These subcodes showed the negative perspectives of interviewees about the accreditation model. The subcode “the difficult and time-consuming model” indicates that accreditation has increased personnel’s responsibility and working pressure, while the approach of quality models, including accreditation, is to make things easier by modifying work processes [18]. This finding is in agreement with the results of Bogh et al. study that examined staff experience and perception of hospital accreditation in Denmark. In that study, the staff described the hospital’s accreditation as meaningless, difficult, and confusing [19].

Based on participants’ opinions, the model increases hospital costs, leads to no adequate outcome, and causes unnecessary documentation and bureaucracy, resulting in a decrease in the quality of services and misunderstanding of the model’s philosophy by the executors in hospitals. These findings are in contrast to those of a study by Jardali et al. in which nurses believed that accreditation is a good tool for improving the quality of hospital care [20] and also disagree with the results of Karimi et al. study that reported the improvement of the quantity of services as a result of the accreditation model [21]. These controversies in results may be due to less attention to proper training and lack of sufficient facilities in military and academic hospitals compared to other hospitals. The results obtained by Brubak et al. indicated no evidence to support accreditation and certification of hospitals linked to measurable changes in quality of care as measured by quality metrics and standards [1].

The results showed that accreditation was identified as a way of money acquisition. The related trainings have caused a source of money acquisition for a group of trainers. Inappropriate provision of training by the MOHME causes hospitals to require additional training to perform accreditation. Trainers also demand large sums of money for training, making this model more difficult to implement, given the adverse budget situation in hospitals, especially in military hospitals.

The interviewees’ negative attitude and hopelessness toward the quality improvement models may be due to ineffectiveness of previous similar models. Furthermore, Alkhenizan and Shaw systematically reviewed health staff attitudes toward accreditation and mentioned negative attitudes of health care professionals about accreditation [22]. Moreover, poor educational policies of the MOHME that have not followed the equality of training programs among hospitals have led to disappointment of the hospitals’ staff. To solve this challenge and improve the executive conditions of these projects, more intersectoral and intrasectoral cooperation between military hospitals and academic hospitals of the MOHME is highly recommended. Nevertheless, the important role of students and faculty members in training hospital personnel should not be disregarded.

The second major code was absence of appropriate executive policy, with 3 subcodes: (1) lack of sufficient personnel and financial resources, (2) disregarding local conditions in implementation and evaluation, (3) and the absence of unity of command in hospitals.

Military hospitals not only suffer from a lack of funds but also suffer from staff shortage. Therefore, the results indicated poor service quality and excessive workload. Numerous studies have indicated that the cost of implementing the accreditation model is high; this is while the validity of the accreditation model, given the costs involved, is in question [23–25]. Also, Bukonda et al. reported specified budget, enough staff, government contribution, and continuous conformity as the requirements of accreditation implementation in developing countries [26].

Since Iran is a vast country, disregarding the local conditions of each regional hospital is another challenge. The MOHME did not consider regional differences or the facilities, infrastructure, and contextual differences in the formulation of the Hospital Accreditation Plan. Pascale Pomey et al. study mentioned attention to environmental differences in hospitals as one of the success factors of the hospital accreditation model [27]. Newhouse et al also mentioned hospital contextual differences as a necessity for improving patient outcomes [28].

The third identified major code was training problems of the accreditation model, with 2 subcodes: (1) absence of proper training and (2) incoordination of training and evaluation. Adequate and complete training is the administrative requirement of each quality improvement system [29]. Soh et al. mentioned that training provided to Malaysian hospitals prior to hospital accreditation led to a significant reduction in hospital infection after accreditation [30]. Difference of training contents with the evaluation criteria caused confusion and concern in the personnel. Moreover, in a study, Amerioun et al. have suggested clear and precise indices and coordination of training and evaluation processes as necessary requirements [31]. In fact, the purpose of accrediting a hospital is to change personnel’s attitudes and improve the work habits of the staff [32].

The other identified code was the problem of human resource, with 3 subcodes: (1) no profit for nonphysician personnel, (2) heavy workload of the personnel, and (3) physicians’ nonparticipation. The accreditation model requires an extended documentation, which increases the workload of the hospital staff and may cause poorer performance. Brubak mentioned that increasing the workload of the personnel in implementing the model was a challenge in his study [1]. Gough et al. showed that a significant number of participants (managers / doctors) found accreditation too bureaucratic, inefficient, and expensive [33].

The accreditation model requires participation of all hospital staff, particularly physicians; however, physicians do not cooperate with its implementation. Baskind et al. found that the accreditation model increased the quality of services and regarded physicians’ cooperation as a significant factor in this success [34]. Alkhenizan & Shaw mentioned that physicians were reluctant to cooperate in the hospital’s accreditation and were suspicious of it [22]. Novaes et al. believed that the accreditation model increases the quality of services and acknowledged physicians’ cooperation as a significant factor in this success [35].

No precise evaluation indicates that evaluators only pay attention to documents without paying attention to the reality of work content in the field that leads to incorrect documentation. Bukonda et al. considered incorrect documentation a sign of accreditation model failure in Zambia [26]. Shaw et al. also argued that proper and precise implementation of the model’s details by personnel guarantees the model success [36].

According to the present study, due to absence of a clear common source for evaluation and evaluators’ different interpretations of indices, there were different perspectives on evaluation, which caused confusion. According to Shaw, a clear homogenous evaluation plays a crucial role in the model success [36]. Changes of evaluation criteria during a short period have caused a change in the whole process of accreditation implementation in the hospital, causing confusion of the involved personnel. All these factors caused poor performance of the studied hospitals regarding implementation of this program. The mentioned finding was not observed in similar studies. However, excessive reliance on certificates without focusing on learning and behavior change leads to several problems, such as incorrect documentation in the process of implementation. Pomey et al. have also emphasized the importance of proper learning of hospitals’ staff in accreditation success [27]. One of the greatest challenges of interviewees was disagreement of training and implementation, which can be overcome by coordinating training and evaluation indices.

Failure to interview the patients as the target group of the accreditation model was a limitation of this study. Thus, interviewing patients could have fulfilled the challenges of implementing the accreditation model.

## Conclusions

This study provided useful data on the challenges of implementing the accreditation model in hospitals. The findings of this study can be used by health policymakers to revise and modify the accreditation procedures in Iran and other countries with similar conditions.

The accreditation model is comprehensive and has been implemented with the aim of improving the quality of services and patient safety. The basic philosophy of hospital accreditation did not fully comply with the underlying conditions of the hospitals. The hospital staff considered accreditation as the ultimate goal rather than a means for achieving quality of service. The MOHME performed accreditation hastily for all Iranian hospitals, while the hospitals were not prepared and equipped to implement accreditation. Also, military hospitals need more attention from the MOHME. Thus, the MOHME should consider these contextual conditions before developing quality models for Iranian hospitals.

## Supplementary information

**Additional file 1.** Interview Guide.

## Data Availability

The interview data used and analyzed during the present study are available in Persian language from the corresponding author upon request.

## Abbreviations

JACHO: Joint Commission on Accreditation of Healthcare Organizations
ACHS: Australian Council of Healthcare Standards
MOHME: Ministry of Health and Medical Education
