# Supplementary material for: Challenges of implementing the accreditation model in military and university hospitals in Iran: a qualitative study
Source: BMC Health Serv Res. 2020 Jul 29;20:698. doi: 10.1186/s12913-020-05536-4 (PMC7392663; doi:10.1186/s12913-020-05536-4)
Supplement: Supplementary file 1 — Additional file 1. Interview Guide. [file 12913_2020_5536_MOESM1_ESM.docx]

**Additional File 1**

**Interview Guide:**

**Introduction:**

**Dear hospital staff:**

You will be invited to participate in a research entitled “Challenges of Implementing the Accreditation Model in Military and University Hospitals in Iran”.

The purpose of this short report is to help you understand why this research is being conducted and what the steps are. If you need more information, please contact me (Nadia Oroomiei). Please read the form carefully and then decide to participate in this research.

**Research Team:**

**Leila Vali**: assistant professor of health services management in faculty of management and medical informatics, Kerman University of Medical Sciences

**Mohammad Hossein Mehrolhasani**: associate professor of health services management in faculty of management and medical informatics, Kerman University of Medical Sciences.

**Saeid Mirzaei**: assistant professor of health policy, Department of Health Management, Policy and Economics, School of Public Health, Bam University of Medical Sciences.

**Nadia Oroomiei**: assistant professor of health policy, Department of Health Management, Policy and Economics, School of Public Health, Bam University of Medical Sciences.

**What is the purpose of this research?**

The purpose of the study is to identify the executive challenges of hospital accreditation in academic and military hospitals.

**Why was I selected to participate in this study?**

Your participation in this study is considered very important due to your valuable knowledge, background and experience in performing accreditation in the hospital, so you have been invited to this study. You don't have to answer if you don't want to.

**What are the steps for participation in this research?**

If you would like to participate in the study, you will need to express your consent to participate in this study. Then about meeting time is coordinated with you. It should be noted that in this meeting, I am going to interview you, which will last about an hour, and the interview will be recorded digitally to facilitate the implementation of the content. Due to your convenience, the interview will be held at your office.

**What are the benefits of participating in this research?**

Although participating in this study may not be of any direct personal benefit to you, your presence in this study and the use of your valuable experience will certainly help to find the implementation challenges of this national program at the hospital level.

**What are the potential harms or risks involved in this research?**

You will not be aware of any potential risks or harms of participating in this research. The information you provide to the researcher is considered completely confidential and no one other than the research team whose names are listed above will have access to this information. You will also be assured that no participant's name will be reflected in any of the published reports or results of this study.

**Does this research have been approved by the relevant authorities?**

This research has been approved by the Research Vice Chancellor of Kerman University of Medical Sciences.

**This information paper belongs to you and you can keep it. Thank you for taking your time and for your cooperation.**

Nadia Oroomiei is assistant professor of health policy, Department of Health Management, Policy and Economics, School of Public Health, Bam University of Medical Sciences. Address: Bam University of Medical Sciences, Shahid Rajaee Blvd., Bam, Iran. . E-Mail: [n_oroomiei@yahoo.com](mailto:n_oroomiei@yahoo.com). Tell: 09216423932

**Interview Questions:**

1. As you know for a while the accreditation model has been implemented in hospitals, how did the accreditation model introduce to the hospital staff?

2. What is your perspective about the model?

3. What are the challenges you have faced while implementing the accreditation model in your hospital during the times?

4. As this is a military hospital, does being a military hospital pose challenges for implementing the model? If yes what are the challenges? (Only ask from military hospital staff)

5. Is there anything else you'd like to talk about?
